# Global Long Noncoding RNA and mRNA Expression Changes between Prenatal and Neonatal Lung Tissue in Pigs

**DOI:** 10.3390/genes9090443

**Published:** 2018-09-05

**Authors:** Long Jin, Silu Hu, Teng Tu, Zhiqing Huang, Qianzi Tang, Jideng Ma, Xun Wang, Xuewei Li, Xuan Zhou, Surong Shuai, Mingzhou Li

**Affiliations:** 1 Institute of Animal Genetics and Breeding, College of Animal Science and Technology, Sichuan Agricultural University, Chengdu 611130, China; longjin8806@163.com (L.J.); erichu121@foxmail.com (S.H.); jaytuteng@163.com (T.T.); wupie@163.com (Q.T.); jideng_ma@sina.com (J.M.); wangxun99@163.com (X.W.); xuewei.li@sicau.edu.cn (X.L.); zhouxuan198866@163.com (X.Z.); 2 Key Laboratory for Animal Disease-Resistance Nutrition of China Ministry of Education, Institute of Animal Nutrition, Sichuan Agricultural University, Chengdu 611130, China; zqhuang@sicau.edu.cn

**Keywords:** long non-coding RNA, mRNA, pig, lung, fetal, neonatal

## Abstract

Lung tissue plays an important role in the respiratory system of mammals after birth. Early lung development includes six key stages, of which the saccular stage spans the pre- and neonatal periods and prepares the distal lung for alveolarization and gas-exchange. However, little is known about the changes in gene expression between fetal and neonatal lungs. In this study, we performed transcriptomic analysis of messenger RNA (mRNA) and long noncoding RNA (lncRNA) expressed in the lung tissue of fetal and neonatal piglets. A total of 19,310 lncRNAs and 14,579 mRNAs were identified and substantially expressed. Furthermore, 3248 mRNAs were significantly (FDR-adjusted *p* value ≤ 0.05, FDR: False Discovery Rate) differentially expressed and were mainly enriched in categories related to cell proliferation, immune response, hypoxia response, and mitochondrial activation. For example, *CCNA2*, an important gene involved in the cell cycle and DNA replication, was upregulated in neonatal lungs. We also identified 452 significantly (FDR-adjusted *p* value ≤ 0.05) differentially expressed lncRNAs, which might function in cell proliferation, mitochondrial activation, and immune response, similar to the differentially expressed mRNAs. These results suggest that differentially expressed mRNAs and lncRNAs might co-regulate lung development in early postnatal pigs. Notably, the *TU64359* lncRNA might promote distal lung development by up-regulating the heparin-binding epidermal growth factor-like (HB-EGF) expression. Our research provides basic lung development datasets and will accelerate clinical researches of newborn lung diseases with pig models.

## 1. Introduction

Lung tissue plays a vital role in gas-exchange and is one of the most important parts of the respiratory system for mammalians [1,2,3]. The six critically important stages of normal lung development are: organogenesis, pseudoglandular, canalicular, saccular, alveolization, and microvascular maturation [4,5,6]. The saccular stage, often defined in gestational weeks 24–36 and can even last until the fourth to fifth year in human postnatal life, is critical for normal lung development because it prepares the distal lung for subsequent alveolarization and gas-exchange after birth, and contributes to the expansion of the alveoli and saccular spaces [6,7]. Before birth, the lung is liquid-filled [8,9,10] and takes no part in gas-exchange because of high pulmonary vascular resistance and immature respiratory function [11,12]. After birth, the liquid is cleared to allow the entry of air and the lung starts to function as an efficient system for gas-exchange [13]. Therefore, it is necessary for the lung to be sufficiently developed at birth to perform the function of gas-exchange, which requires numerous physiological changes to occur [14]. However, little is known about changes in messenger RNA (mRNA) and long non-coding RNA (lncRNA) expression during this transition [15,16].

Pigs (*Sus scrofa*) have many similarities with humans including comparable visceral organ size, and similar skin, respiratory and digestive tracts, and immune systems [17,18]. Thus, the pig is an ideal animal model for human pathologies. Indeed, researches of several respiratory pathologies including cystic fibrosis [19,20], lung transplantation [21], and influenza A virus infection [22] often use pigs as models.

Long non-coding RNA are defined as non-protein coding transcripts longer than 200 nucleotides [23]. Initially thought to be transcriptional noise, lncRNAs are involved in major mechanisms of gene expression, regulation, and cellular development [24]. To explore the mechanisms and the transcriptomic expression profiles of mRNA and lncRNA in the lung, we performed RNA-sequencing (RNA-seq) analysis on fetal (the last day of perinatal) and neonatal (two days postnatal) piglet lungs. We identified 3248 differentially expressed (DE) mRNAs, which were mainly enriched in the categories of cell proliferation, immune response, and mitochondrial activation, which may be the consequence of the lung’s adaptation to air-breathing after birth. Interestingly, we found that the lncRNA *TU64359* might promote distal lung development by up-regulating the expression of heparin-binding epidermal growth factor-like expression (HB-EGF), which is critical for normal lung development and has a suppressive function that synergistically contributes to decelerating hypercellularity in the distal lung with transforming growth factor-α (TGFα) through epidermal growth factor receptor (EGFR) in perinatal distal lung development [25].

## 2. Materials and Methods 

### 2.1. Animals and Samples

Six female Tibetan-Meishan (Patriarchal: Tibetan pig; Matriarchal: Meishan pig) hybrid piglets were used to simulate the adaptation of newborns. The pregnancy period was considered to be 113 days. The fetal group consisted of three piglets obtained from three pregnant sows of similar weights on the last day before birth. The neonatal group consisted of three piglets of similar weight obtained two days after birth from another three pregnant sows. All parents were of a similar-weight and were raised under the same feeding and rearing conditions until caesarean and parturition. Piglet lung tissues were rapidly sampled from each carcass and selectively separated into two parts. One part was fixed with 10% formalin for morphometric analysis, and the other part frozen immediately in liquid nitrogen and stored at −80 °C until required for RNA extraction.

All the pigs were provided by the Sichuan Animal Science Academy, Chengdu, China and were sacrificed as necessary to ameliorate suffering by exsanguination following ketamine (20 mg/kg) intramuscular injection. All experimental and sample collection procedures were approved by the Institutional Animal Care and Use Committee (IACUC) of the College of Animal Science and Technology of Sichuan Agricultural University, Sichuan, China under permit No. DKY-B20171902.

### 2.2. Morphometry of Lung Tissues

Lung tissues were fixed at 4 °C with 10% formalin overnight. Then, the samples were embedded in paraffin and sliced into 4 mm thick sections. Motic Images Advanced v.3.2 (Motic, Hong Kong, China) was used to analyze the structure of the lungs at 100× and 400× magnification after staining of each slice with hematoxylin and eosin (H&E staining). The steps of H&E staining were: (1) Dip slices into 95% ethanol for 5 to 10 min; (2) dip slices into 85% ethanol, then 75% ethanol (in distilled water) for 5 min in turn; (3) wash each slice slightly after staining for 10 to 15 min in hematoxylin; (4) differentiation in 0.5% chlorane for 3 s, and dipped into distilled water for 15 to 20 min; (5) stain in eosin for 10 min after 5 min dehydration in 75%, 85%, and 95% ethanol by turn; (6) wash slices with 95% ethanol and dehydrate for 30 min in 100% ethanol; (7) extract the alcohol with two changes of xylene for 20 to 30 min; and (8) one or two drops of neutral resins and cover with a coverslip.

### 2.3. RNA Isolation, Library Preparation, and Sequencing

Total RNA was isolated for each sample with Trizol reagent (Life Technologies, Beijing, China) according to the manufacturer’s instruction. The integrity and quality of total RNA samples were analyzed with an RNA 6000 Nano kit (Agilent Technologies, Palo Alto, CA, USA) on a Bioanalyzer 2100 system. Approximately 1 µg total RNA and the Ribo-Zero™ kit (Epicentre, Madison, WI, USA) were used to generate RNA-seq complementary DNA (cDNA) libraries for each sample, and then, RNA sequencing was performed following the manufacturer’s standard procedures. Complementary DNA size and purity were checked using a high sensitivity DNA 1000 kit and AMPure XP beads and was amplified using PCR. High-quality strand-specific libraries were sequenced on the HiSeq X Ten platform (Illumina, San Diego, CA, USA) and the bases were called using the software CASAVA v.1.8.2 (Illumina), then the pair-end reads were acquired.

To avoid artificial bias, we removed low-quality reads including those with ≥10% unidentified nucleotides, >10 nt aligned to the adapter with ≤10% mismatches allowed, and with >50% of bases with phred quality <5. Consequently, 86.46 Gb data was retained, of which the quality of 90% of the base ≥Q30 (Table 1). All RNA-seq data have been deposited in NCBI’s (National Center for Biotechnology Information) Gene Expression Omnibus (GEO) series accession number GSE117882.

### 2.4. Messenger RNA Expression Analysis

High-quality reads of six strand-specific libraries were mapped to the pig reference genome (*Sus Scrofa* 10.2 from Ensembl) with TopHat v.2.1.0 [26] with parameter of—*library-type* = *fr-firststrand* (other parameters in default). Messenger RNA expression levels of fragments per kilobase per million mapped reads (FPKM) were obtained using Cufflinks v.2.2.1 [26] and mRNAs with FPKM >0.5 in at least one sample were considered to be expressed. Then, Cuffquant (part of Cufflinks) was used to generate abundance files, which were applied to Cuffdiff (part of Cufflinks) to detect DE mRNAs/genes between the two groups. The parameters of Cufflinks/Cuffquant/Cuffdiff were set to—*library-type* = *fr-firststrand, -u, -b/-G* according to the corresponding utilities. To evaluate global mRNA expression changes in these two developmental stages mRNA/genes with *q* (FDR-adjusted *p*) value ≤ 0.05 were considered to be DE genes.

### 2.5. Long Non-Coding RNA Identification and Expression Analysis

To obtain lncRNAs transcripts, the mapped reads for each sample were assembled using Stringtie v.1.2.2 [27]. Then, AssemblyLine [28] was used to characterize and filter background noise and to perform meta-assembly [24] by merging the assembled transcripts. Coffcompare (part of Cufflinks) was used to remove transcripts annotated in the reference sequence (marked by ‘c’ for partial match or ‘=’ for full match). Next, transcripts with lengths greater than 200 nt were used for the prediction of coding potential according flowed steps: (1) Each transcript sequence was translated in all six possible frames with Transeq (part of EMBOSS v.6.5.7) [29] and the transcript with translated protein sequences that had a significant hit in the Pfam (release27) database [30] with HMMER v3.1b2 [31] were excluded. (2) Using BLASTX (https://blast.ncbi.nlm.nih.gov/), the remaining transcripts were compared with human and mouse genomes, and the UniRef database (https://www.uniprot.org/), and potential coding transcripts were removed. (3) The CPC (coding potential calculator) [32] was used to assess coding potential in both strands of the remaining sequences and those with a negative CPC score (noncoding) were kept. (4) Lastly, we quantified the remaining transcripts and obtained FPKM values using Cufflinks. Only transcripts with FPKM > 0.1 in at least two replicates of one group were used for further analysis. The steps of lncRNA identification are depicted in Figure S1.

The lncRNA.gtf file, which was created through the described steps and contained lncRNA transcript informations, was merged with the reference annotated pig-genome-annotated.gtf file to create a new reference annotated lncRNA-merged-pig-genome-annotated.gtf file. We then used Cuffquant and Cuffdiff to detect DE lncRNAs, those lncRNAs with *q* value ≤ 0.05 DE lncRNAs.

### 2.6. Long Non-Coding RNA Classification

To obtain genomic characterizations for all identified lncRNA transcripts, we ran FEELnc_classifier.pl (a Perl script in FEELnc v.3) [33]. Transcripts with an *isBest* (*isBest*: best interactions between lncRNA and nearest genes) value = 1 were considered to be orientated lncRNAs. These lncRNAs were then classified into five classes according to the FEELnc output of the corresponding gene. (1) lincRNAs (intergenic lncRNAs) are lncRNAs located between annotated protein-coding genes and at least 1 kb away from the nearest protein-coding genes [34]. (2) Antisense lncRNAs are transcribed from the antisense strand and overlap, in part, with well-defined sense RNAs [35]. (3) Sense-overlapping lncRNAs overlap with a known annotated gene on the same genomic strand and could be considered as transcript variants of protein-coding mRNAs [36]. (4) Divergent lncRNAs are oriented head to head with a protein-coding gene within 1 kb. (5) Convergent lncRNAs are opposite to divergent lncRNAs and are tail to tail with a protein-coding gene within 1 kb [37].

### 2.7. Functional Enrichment Analysis

To predict the functions of the identified lncRNAs, we extracted highly related mRNAs for them. First, we collected cis nearby mRNAs within 100 kb upstream and downstream of the lncRNAs. Then, *Hmisc* (an R package from https://cran.r-project.org/) was applied to calculate Pearson correlations between the lncRNAs and the mRNAs. Messenger RNAs with high correlations (|*r|* > 0.95 and *p* value < 0.05) were collected. These mRNAs were defined as highly-related ones which may have some similar structures or functions to the lncRNAs.

Differentially expressed mRNAs and the highly-related genes for DE lncRNAs were respectively assessed by the DAVID [38] webserver for functional enrichment in gene ontology (GO) terms including molecular function, cellular components, and biological processes as well as the Kyoto Encyclopedia of Genes and Genomes (KEGG; https://www.genome.jp/kegg/) pathways. For these functional enrichment analyses, we correspondingly set the DE mRNAs as gene list and all tested mRNAs as background.

### 2.8. Quantitative Real-Time PCR Validation

Twenty-two DE genes (Table S1) were selected to verify the expression changes observed in our RNA-seq results. Primers (with two internal control genes of *GAPDH* and *TOP2B*) were designed with software Primer Premier 5.0 and checked with Primer-BLAST [39] of NCBI, and then synthesized by BGI-Shenzhen (BGI-Shenzhen Co., Shenzhen, China). After the melting temperature (Tm) value was confirmed with pre-experiments, 1 µg total RNA was reverse transcribed using M-MLV (Moloney Murine Leukemia Virus) RNAse H-negative reverse transcriptase (Takara Biotechnology Co., Dalian, China). Then, we performed qRT-PCR (Quantitative Real-Time PCR) with SYBR^®^ Green Real-Time PCR Master Mix (Takara Biotechnology Co., Dalian, China) using a CFX96™ Real-Time PCR Detection system (Bio-Rad Co., Hercules, CA, USA) according to the manufacturer’s protocol. 

We also performed a negative control, with sterile water and no cDNA template, for each measurement. The qRT-PCR measurements were performed with three replicates for each sample. *GAPDH* and *TOP2B* were simultaneously used as endogenous control genes. The expression level changes in the surveyed samples were determined by the 2^−ΔΔCt^ method and *t*-test.

## 3. Results

### 3.1. Morphologically Differences between Fetal and Neonatal Pigs

**Figure 1:**
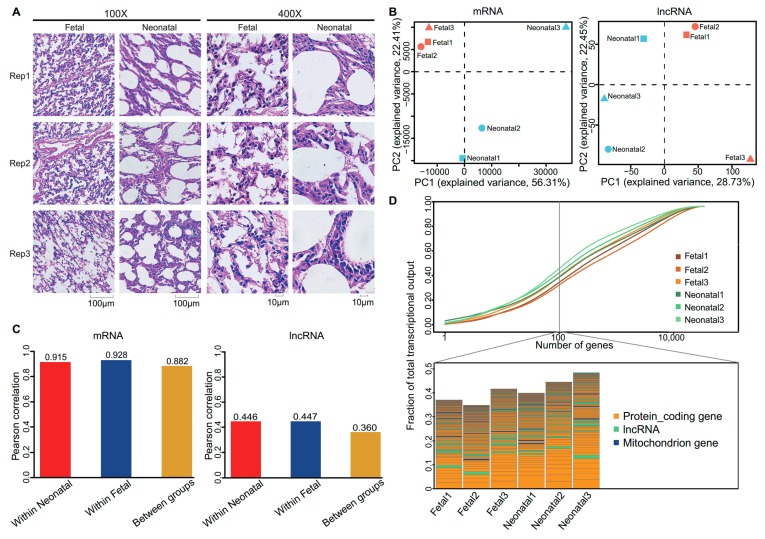
(**A**) Magnified images of the lungs in fetal and neonatal groups (Left: 100×; Right: 400×). Rep1, rep2, and rep3 are representative of tripartite biological replicates. (**B**) Principal component analysis (PCA) was performed on the expression levels of messenger RNA (mRNA) and long non-coding RNA (lncRNA) with log_2_-transformed FPKM (fragments per kilobase per million mapped reads) values. (**C**) Pearson correlation coefficients between samples in the same group or between two groups. (**D**) The complexity of total (mRNA and lncRNA) transcripts. Top: Cumulative measures of the fraction of total transcripts contributed by mRNA and lncRNA (all transcripts were sorted from most-to-least). Bottom: Biological type and relative contribution to total transcripts of the top-100 expressed genes or transcripts. Height of the bar shows the relative contribution of transcripts to the total. PC: Principal component.

Our study revealed significant morphological differences between the lungs of fetal and neonatal pigs. In the fetal group, consistent with their liquid-filled condition before birth, the alveoli were compressed and alveolar boundaries were difficult to clearly recognize [40,41,42] as revealsed in Figure 1A. In contrast, the alveolar boundaries of the neonatal group were trenchant and smooth and the alveoli size was larger than that observed in the fetal samples. Morphological changes in neonatal lungs were consistent with decreased liquid pressure as a result of liquid clearance after birth [40,41,42,43].

### 3.2. Expression Profiles of Messenger RNA and Long Non-Coding RNA in Lung 

For the six Ribo-zero RNA sequencing libraries, we yielded approximately 102.86 million 150-bp paired-end reads. After filtering, we acquired ~86.46 Gb (~14.41 Gb per sample) of high-quality data, and approximately 65.89 Gb (10.98 Gb per sample) were mapped to the pig reference genome (Table 1).

In total, 14,579 mRNAs were expressed (FPKM > 0.5 in at least one sample), and 19,310 lncRNAs were substantially expressed (FPKM > 0.1 in at least two replicates of one group). Messenger RNA and lncRNA expression varied greatly between the fetal and neonatal groups. Principal component analysis (PCA) shows that the six pigs can be clearly differentiated at both mRNA and lncRNA expression levels (Figure 1B). The average Pearson correlation coefficient of mRNAs within each group was higher than that of lncRNAs (Figure 1C), this may reflect differences in natural expression variability between mRNA and lncRNAs, as lncRNAs always have a higher natural expression variability of mRNAs [44]. The different expression profiles can also be indicated by heatmaps with hierarchical clustering (Figure S2A,B) and pairwise Pearson correlation coefficients (Figure S2C,D). These results suggest that there is a high correlation between the gene expression levels of the biological replicates and show that our experiment has good reliability.

The transcriptional complexity of the neonatal group was slightly lower than that of the fetal group, with the top 100 highly expressed genes in the neonatal group accounting for a higher fraction of total transcripts (Figure 1D). In particular, the expression of many mitochondrial genes were raised in the neonatal group (Table 2). This may be a coincidence as the timing corresponds with the mitochondrion activation and energy metabolism (discussed below). These results suggest that transcription is generally dominated by the expression of a relatively small number of genes, and is consistent with the expression profiles of many human tissues [45].

### 3.3. Characteristics Comparison of Messenger RNAs and Long Non-Coding RNAs

Characteristics comparison of mRNA and lncRNA revealed that approximately 90% of the lncRNA transcripts contain 1 or 2 exons, and that more than 80% of the mRNA transcripts had ≥3 exons (Figure S3A). The length of most (75.83%) of the lncRNAs was between 200 and 1000 bp, and the length of most (67.70%) mRNAs was between 500 bp and 3000 bp (Figure S3B). The median length (1767 bp) of mRNA was much longer than that (574 bp) of lncRNA. Our results also suggest that there were lower expression and coding potentials in lncRNA (Figure S3C,D). These results were coincident with the previous study which concluded that lncRNAs generally have lower expression levels than the transcripts of protein coding genes [46].

### 3.4. Functional Enrichment Analysis of Differentially Expressed Messenger RNAs and Long Non-Coding RNAs

**Figure 2:**
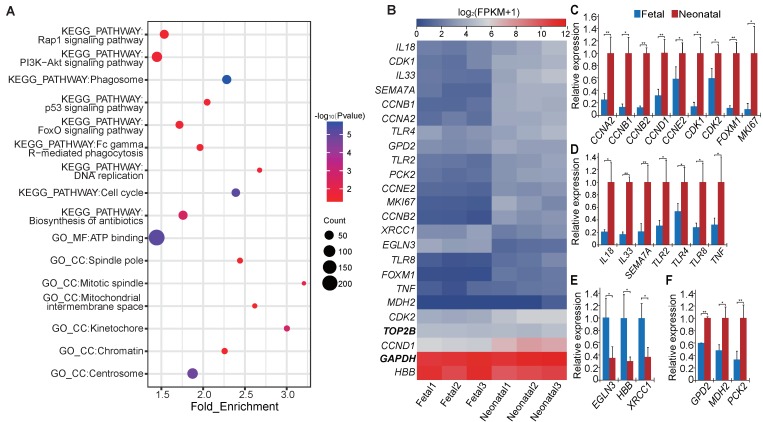
Differentially expressed (DE) mRNAs revealed the changes in the lung after birth. (**A**) Mainly enriched categories for differentially expressed genes. The function enrichment analysis of the DE genes was performed using DAVID and the listed terms were with *Benjamini* corrected *p* value < 0.05; we set 3248 DE genes as gene list and all expressed genes (14,579 mRNAs) as background. (**B**) Heat map of 22 selected DE genes (GAPDH* and TOP2B* were used as internal control genes). Quantitative Real Time-Polymerase Chain Reaction (qRT-PCR) expression verification of crucial genes in the following related categories: (**C**) cell proliferation, (**D**) immune response, (**E**) hypoxia response, (**F**) and mitochondrial activation. * *p* value < 0.05, ** *p* value < 0.01.

In total, 3248 DE mRNAs were obtained in our study (Table S2). Functional enrichment analysis revealed that most of these DE mRNAs were mainly enriched in categories (*Benjamini* corrected *p* value < 0.05) related to cell proliferation, immune response, and mitochondrial activation (Figure 2A and Table S3). We even found some DE mRNAs enriched in several categories (*Benjamini* corrected *p* value < 0.05), such as *Rap1*, *PI3K−Akt*, *p53,* and *FoxO* signaling pathways which were critical in the regulation of cell proliferation and apoptosis, individual development and other biological processes. Besides, a category of hypoxia response (*p* < 0.05) was protruded. Previous studies showed that some of the significantly DE genes (Figure 2B) identified in this research played important roles. 

Some of the DE genes that related to cell proliferation were highly increased after birth. For example, *CCNE2* (log_2_(FC) = 1.90, *q* value = 5.15 × 10^−3^), *CDK2* (log_2_(FC) = 1.21, *q* value = 6.53 × 10^−4^), and *CCND1* (log_2_(FC) = 1.59, *q* value = 6.53 × 10^−4^) play key roles in the cell cycle during the G1/S transition. Overexpression of *CCND1* in fibroblasts could accelerate G1 progression, and *CCND1* inhibition prevents cells from entering S-phase [47,48,49,50]. *CCNA2* (log_2_(FC) = 2.34, *q* value = 6.53 × 10^−4^) is especially active in the S-phase, and could regulate the S/G2 transition and DNA replication [51,52,53]. *CCNB1* (log_2_(FC) = 3.10, *q* value = 6.53 × 10^−4^) and *CCNB2* (log_2_(FC) = 3.37, *q* value = 1.64 × 10^−3^) promote the G2/M phase transition, and *CCNB2* can activate *CDK1* (log_2_(FC) = 2.14, *q* value = 6.53 × 10^−4^) [54], which is crucially important in several phases of mitosis [55,56]. Furthermore, *FOXM1* is another critical cell cycle gene which can activate proliferation and enhance β-cell proliferation [57,58]. In addition, the KI67/MKI67 (log_2_(FC) = 3.50, *q* value = 6.53 × 10^−4^) proliferation marker gene [59] was also significantly increased in the neonatal group.

For DE genes related to immune response, the Toll-like receptor (TLR) genes including *TLR2* (log_2_(FC) = 1.55, *q* value = 6.53 × 10^−4^), *TLR4* (log_2_(FC) = 0.91, *q* value = 3.30 × 10^−3^), and *TLR8* (log_2_(FC) = 2.12, *q* value = 6.53 × 10^−4^) and some respiratory-related genes such as *IL18* (log_2_(FC) = 1.65, *q* value = 1.18 × 10^−3^), *IL33* (log_2_(FC) = 2.60, *q* value = 6.53 × 10^−4^), *TNF* (log_2_(FC) = 1.23, *q* value = 4.81 × 10^−2^)*,* and *SEMA7A* (log_2_(FC) = 3.06, *q* value = 6.53 × 10^−4^) were significantly highly expressed in neonatal lungs. Previous studies showed that these TLRs are expressed in airway epithelial cells and participate in the recognition of microorganisms [60], lipopolysaccharide [60,61], and in the innate antiviral response [62]. Moreover, *IL18*, a member of the IL-1 family, can regulate both innate and acquired immune responses, and is related to acute lung inflammation [63,64]. *IL33* is expressed in airway epithelial cells and plays a role in the maturation and function of dendritic cells [65]. *TNF* can enhance phagocytosis of phagocytes, and over-expression of *TNF* heightens the clearance of bacteria in *Klebsiella pneumoniae* pneumonia [66]. *SEMA7A* stimulates monocytes and macrophages to secrete cytokines and is important for inflammatory immune responses. Decreased *SEMA7A* expression in mice causes cell-mediated immune response deficiencies [67].

Among the DE genes related to hypoxia response, *EGLN3* (log_2_(FC) = −2.88, *q* value = 6.53 × 10^−4^) is a typical hypoxia-inducible gene that can regulate the hypoxia-inducible factors (HIF) transcriptional pathway [68]. *XRCC1* (log_2_(FC) = −0.77, *q* value = 3.30 × 10^−3^) could mediate the repair of DNA-damage caused by acute phase of asphyxia [69]. Moreover, *HBB* (log_2_(FC) = −1.17, *q* value = 6.53 × 10^−4^) and *HBA* are important components of hemoglobin [70]. These hypoxia-response-related genes were all increased significantly in fetal lungs.

For DE genes related to mitochondrial activation or energy metabolism, *MDH2* (log_2_(FC) = 0.89, *q* value = 6.53 × 10^−4^) encodes malate dehydrogenase and other mitochondrial enzymes, and is crucial in the tricarboxylic acid (TCA) cycle [71]. *GPD2* (log_2_(FC) = 0.74, *q* value = 4.80 × 10^−3^), which is important in sustaining glycolysis, could also encode some mitochondrial enzymes [72]. *PCK2* (log_2_(FC) = 1.90, *q* value = 6.53 × 10^−3^) encodes mitochondrial phosphoenolpyruvate carboxykinase, which links glycolytic and TCA cycle intermediates [73]. These genes that promote energy metabolism were all significantly upregulated in neonatal group.

DE genes mentioned above were validated by qRT-PCR (Figure 2C–F).

**Figure 3:**
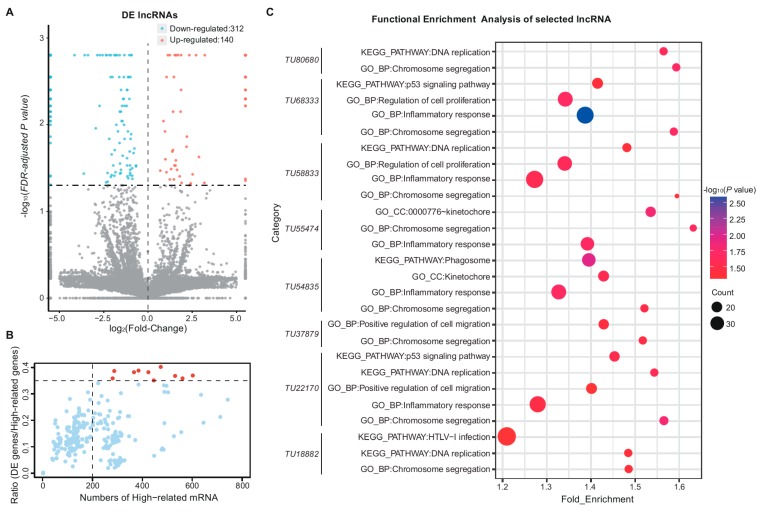
DE lncRNAs revealed the changes in the lung after birth. (**A**) DE lncRNAs with *q* value ≤ 0.05: 312 were down-regulated, 140 were up-regulated; (**B**) DE lncRNAs distribution based on the ratio of DE genes per highly-related genes and the total highly-related mRNA number. Highly related genes were identified by comparison between mRNAs and lncRNAs as described in methods. A total of 136 lncRNAs had more than 200 related genes and only 10 lncRNAs (marked in red) with a ratio (DE genes per highly-related genes) ≥0.35; (**C**) Functional enrichment analysis of the top ten lncRNAs in (**B**) with their related genes, the highly related DE genes were used as gene list while the highly related genes were set as background. Categories (*p* value < 0.05) related to cell proliferation and immune response were listed.

Our research also identified 452 DE lncRNAs (Figure 3A and Table S4). Of them, 140 lncRNA transcripts were upregulated and 312 lncRNA transcripts were downregulated in the lung after birth. To predict the function of these DE lncRNAs, we analyzed a total of 768 (171 were DE genes) genes situated within 100 kb upstream or downstream of them, and 7318 (2924 were DE genes) highly correlated genes (|*r|* > 0.95 and *p* < 0.05). Among these highly related mRNAs, there were only 365 (153 were DE genes) genes with a high correlation in nearby loci. After functional enrichment analysis of the 153 DE genes with setting all related genes as background, only few categories (*p* < 0.05) related to the regulation of some biological process (Table S5).

In addition, we found 136 lncRNAs (30% of DE lncRNAs), each of which had more than 200 highly related mRNAs, and only 10 lncRNAs with a ratio (DE genes per highly-related genes) ≥35% (Figure 3B). On the basis of the functional enrichment analysis of the top ten ratio-ranked lncRNAs (*TU37879*, *TU15422*, *TU72191*, *TU18882*, *TU54835*, *TU58833*, *TU22170*, *TU55474*, *TU68333*, and *TU80680*), we found many of them fell in categories (*p* value < 0.05) related to cell proliferation and immune response (Figure 3C). 

### 3.5. Functional Enrichment Analysis of Different Long Non-Coding RNA Types

**Figure 4:**
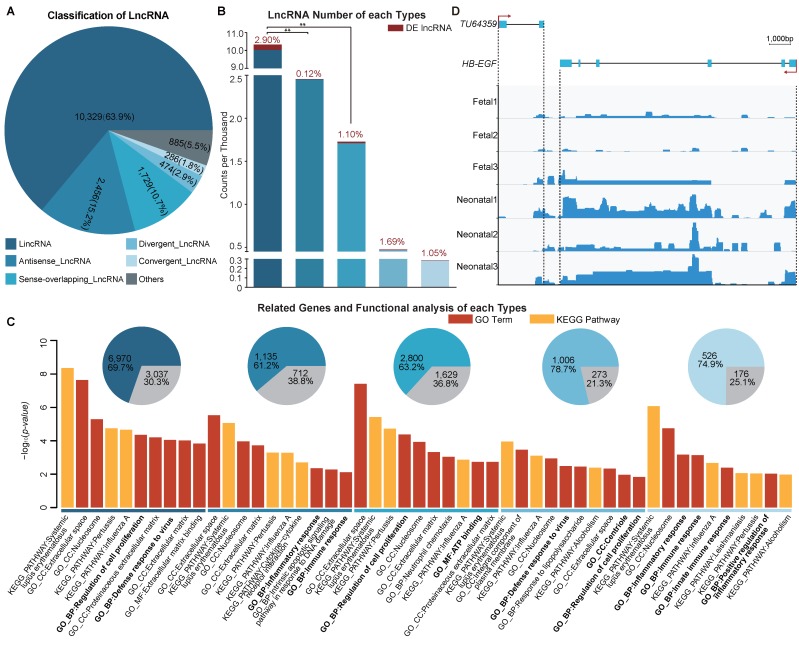
Classification and functional enrichment analysis of lncRNAs. (**A**) Classification of lncRNAs: proportion and number of each type is shown in the pipe plot; (**B**) lncRNA number for the different types: bars represent the counts for different types, DE lncRNAs numbers and ratios are labeled in red, χ-squared tests were performed on the counts between lincRNAs with other types. (**C**) Related genes and functional enrichment analysis of different lncRNA types: highly related genes for DE lncRNA of different types shown in pie plots (DE mRNAs are labeled in gray), top ten categories (*p* < 0.05) are listed in bar plots after enrichment analysis by DAVID, DE mRNAs were set as gene list and all related genes as background. Categories related to cell proliferation, immune response and mitochondrial activation are in bold font; (**D**) Loci and expression levels of *TU64359* and *HB-EGF* read abundance which was generated by integrative genomics viewer (IGV) v.2.4.10 [74]. **: *p* < 0.01.

We separated the 16,159 lncRNAs to five different types (Table S6). LincRNAs accounted for the largest proportion of lncRNAs (10,329, 63.9%) (Figure 4A) and were prone to being differentially expressed. Moreover, the counts of DE lincRNAs was significant higher (*p* < 0.01) than that of antisense lncRNA and sense-overlapping lncRNAs, and showed higher drifts than the other lncRNA types (Figure 4B). As demonstrated in Figure 4C, lncRNAs in one or more types might be related to cell proliferation, immune response, and mitochondrial activation.

Notably, we found a lncRNA (*TU64359*: chr2, 148,043,887:148,046,077) which is a convergent lncRNA of *HB-EGF* (Heparin-binding epidermal growth factor-like growth factor, chr2, 148,046,706:148,058,378), and together with *HB-EGF* might co-regulate perinatal distal lung development (Figure 4D and Table S7). The Pearson coefficient value of this gene/lncRNA pair was 0.85 (*p* value = 0.03) and the genetic distance was 629 bp. Previously, *HB-EGF* has been shown to have a suppressive function and to decelerate distal lung cell proliferation synergistically with *TGFα* through *EGFR* in perinatal distal lung development [25]. Expression levels of *TU64359* and *HB-EGF* were raised significantly in the neonatal group (Figure 4D), which may indicate that *TU64359* might regulate the *HB-EGF* expression levels.

## 4. Discussion and Conclusions

The lung is critical for mammals and is in intimate contact with the vitro environment [75]. Huge amounts of air are passed in and out of the lung every day, which, in humans, amounts to 5 to 8 liters per minute. Previous studies showed that lung tissue goes through an acute transition during the development from the fetal to the neonatal stage, without intrauterine protections, in mammals [76]. Numerous physiological transformations occur to facilitate adaption to the vitro environment. To reveal the mechanisms involved in such transformations and the transcriptome expression profile changes in the lung between the two different stages, we conducted this research.

Before birth, the lungs are in conditions of hypoxia and asphyxia due to lung fluid and limited uterine space, which can result in oxygen-deficiency and DNA-damage [8,9,10,12]. In the present study, *EGLN3*, *XRCC1*, and *HBB* were highly expressed in lungs before birth. This expression profile might facilitate maintaining the balance between oxygen and DNA-damage repair in fetal lungs, which is consistent with functions that have been previously ascribed to these genes [68,69,70].

The saccular period is vital for lung development including alveolarization and saccular space expansion [25], especially in the few days after birth. Lungs increase in size for a significant time after birth as a result of an increase in the length and diameter of the airways [77,78]. The morphological changes described in this study are consistent with this. Moreover, our results showed that some DE genes were enriched in categories related to cell proliferation, which are often highly expressed after birth and function to promote cell proliferation or cell cycle progression. We validated that these genes are highly expressed in neonatal lungs.

The immune system was not fully developed in the examined tissues as the lungs are structurally immature at the time when newborns begin to live in the external environment with various different microorganisms [79]. That is why we found that some genes that may enhance the activity of the immune system presented a significantly increased expression in neonatal lungs, including *TLRs* and *IL-1* family members.

Physical processes in neonatal lungs, such as cell proliferation, changes in pulmonary blood pressure, and immune response require much energy. As expected, there were some DE genes enriched in the categories of mitochondrion activities and energy metabolism. Increased expression of *MDH2*, *GPD2*, and *PCK2*, as observed in neonatal lungs, would enhance mitochondrial activation or promote the TCA cycle [71,72,73] in the lung.

Long non-coding RNAs, non-coding transcripts longer than 200 nucleotides, are emerging as key regulators of expression for some genes and of biologically processes [23]. In the present study, our results indicate that lncRNA might co-regulate normal lung development with mRNAs as the lncRNA-mRNA pair (*TU64359* and *HB-EGF*) shows. Previous studies also revealed interactions between lncRNA and genes in the lung, for example lncRNA *SNHG20* (Small Nucleolar RNA Host Gene 20) and gene NSCLC (non-small cell lung cancer) [80]. *HB-EGF* plays an important role in perinatal normal lung development, consequently HB-EGF null lungs showed abnormally thick saccular walls caused by increased rate of cell proliferation. Therefore, an increased HB-EGF expression level in neonatal lungs may indicate decreased hypercellularity and maintenance of normal development [25]. This could explain the distinct changes in morphometry and different functions of the lung between two phases. However, the detailed mechanisms of the interactions remain unclear and more studies need to be performed to fully elucidate the relationship.

Pigs could be ideal models for many clinical investigations related to humans, but there are still considerable differences in lung development between pigs and humans. Many researches about lung development were performed on humans and mice, which have a relatively clear lung development timeframe [7,25,81]. However, few studies have compared pig lungs to other model animals at the level of alveolar morphology or cells composition [82,83,84], and the definite entire timing phases of porcine lungs remains unclear. Thus, further establishment of the precise timeframe of pig lung development will be needed to promote pigs being an available module for lung development research.

In conclusion, we systematically identified and classified lncRNAs in the lungs of fetal and neonatal pigs using RNA-seq data. Our combination analysis results of mRNA functional enrichment and lncRNA functional prediction suggested that lncRNA might promote lung development through cell proliferation, immune response, and mitochondrial activation by co-regulation with mRNAs. Our study may accelerate research into pig models as regards them being ideal models for clinical research of human newborn diseases.

## Figures and Tables

**Table 1:** Summary of data information.

Sample Name	Raw Data (Gb)	Clean Data (Gb)	Proportion of Q30 (%)	Raw Reads	Clean Reads	Mapped Reads	Map Ratio (%)
Fetal1	15.48	14.60	91.99	103,218,160	97,324,642	74,846,405	76.90
Fetal2	15.07	13.60	91.93	100,467,280	90,697,458	69,307,578	76.42
Fetal3	14.13	13.52	92.14	94,178,310	90,125,098	68,548,684	76.06
Neonatal1	15.53	14.44	91.56	103,521,604	96,233,814	72,838,975	75.69
Neonatal2	16.23	15.41	91.75	108,169,398	102,757,612	78,091,900	76.00
Neonatal3	16.14	14.89	91.84	107,604,186	99,294,804	75,662,550	76.20

**Table 2:** Expression levels of annotated mitochondrial genes.

Gene Name	Fetus 1	Fetus 2	Fetus 3	Neonatal1	Neonatal2	Neonatal3	log_2_(FC) ^2^	*q* Value ^3^
*ATP6*	279.86	325.45	361.37	813.54	312.92	436.18	0.49	5.00 × 10^−1^
*ATP8*	1828.44	1283.11	1824.56	16,615.00	6478.03	4800.99	2.42	2.97 × 10^−1^
*COX1*	1894.03	2303.75	2511.00	2865.40	2338.47	4216.83	0.16	7.36 × 10^−1^
*COX2*	1266.21	1274.47	1569.65	2131.98	1818.49	2814.47	0.36	2.30 × 10^−1^
***COX3*** ^1^	495.71	517.16	667.49	1252.63	778.14	1285.44	0.79	1.85 × 10^−2^
***CYTB*** ^1^	24.26	34.00	41.33	102.45	36.26	41.20	0.82	2.90 × 10^−3^
***ND1*** ^1^	18.68	28.52	36.24	92.43	38.03	60.25	0.97	6.53 × 10^−4^
***ND2*** ^1^	10.90	19.53	22.55	70.53	18.87	35.77	1.21	6.53 × 10^−4^
***ND3*** ^1^	1118.87	923.81	1311.11	2951.35	2004.06	3626.46	0.98	6.53 × 10^−4^
***ND4*** ^1^	15.15	25.23	32.48	89.11	31.83	25.04	0.99	6.53 × 10^−4^
*ND4*	91.18	120.58	141.40	297.58	106.88	40.46	0.29	9.30 × 10^−1^
***ND5*** ^1^	17.82	34.67	35.99	125.60	34.05	30.01	1.24	6.53 × 10^−4^
*ND6*	8.56	3.81	28.28	11.18	4.98	14.80	−0.78	8.50 × 10^−1^

^1^ Mitochondrial genes in bold fonts had significantly raised the expression levels in neonatal lungs; ^2^ log_2_(FC): log_2_(Fold Change); ^3^ *q* value: FDR-adjusted *p* value.

## Supplementary Materials

The following are available online at http://www.mdpi.com/2073-4425/9/9/443/s1. Figure S1: Strategy for lncRNA identification. Figure S2: The FPKM value of mRNA and lncRNA were transformed by log_2_(FPKM+1). This information was used to draw heatmaps with hierarchical clustering and pairwise Pearson correlation coefficients. Heatmaps for hierarchical clustering (A: mRNA, B: lncRNA) and pairwise Pearson correlation coefficients (C: mRNA, D: lncRNA). Figure S3: Comparison of mRNA and lncRNA characteristics. (A) Exon number (transcripts with 1–15 exons were included), (B) length of transcripts, (C) expression level (FPKM value transformed by log_10_(FPKM+1)), and (D) coding potential (CPC value for mRNA was calculated by CPC either). Table S1: Primer sequences of selected genes. Table S2: DE mRNAs and their expression in each sample. Table S3: Funtional enrichment analysis of DE genes. Table S4: DE lncRNAs and their expression in each sample. Table S5: Functional enrichment analysis of DE genes in high correlation and nearby loci. Table S6: lncRNA classifications. Table S7: Loci informations of the lncRNA-mRNA pair.
