# Supplementary material for: Global Long Noncoding RNA and mRNA Expression Changes between Prenatal and Neonatal Lung Tissue in Pigs
Source: Genes (Basel). 2018 Sep 5;9(9):443. doi: 10.3390/genes9090443 (PMC6162397; doi:10.3390/genes9090443)
Supplement: Supplementary file 1 [file genes-09-00443-s001.zip › Fig.S1.pdf]

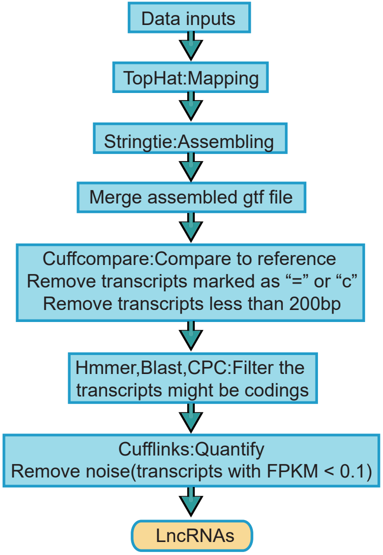
Data inputs
TopHat:Mapping
Stringtie:Assembling
Merge assembled gtf file
Cuffcompare:Compare to reference
Remove transcripts marked as “=” or “c”
Remove transcripts less than 200bp
Hmmer,Blast,CPC:Filter the
transcripts might be codings
Cufflinks:Quantify
Remove noise(transcripts with FPKM < 0.1)
LncRNAs
