# Supplementary figures and images for: Global Long Noncoding RNA and mRNA Expression Changes between Prenatal and Neonatal Lung Tissue in Pigs
Source: Genes (Basel). 2018 Sep 5;9(9):443. doi: 10.3390/genes9090443 (PMC6162397; doi:10.3390/genes9090443)

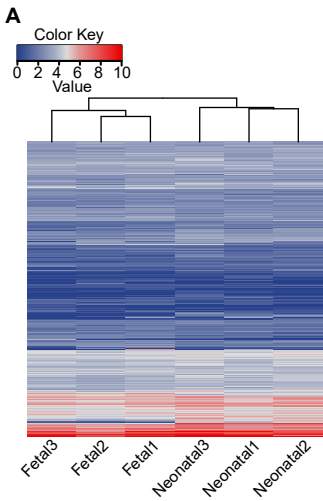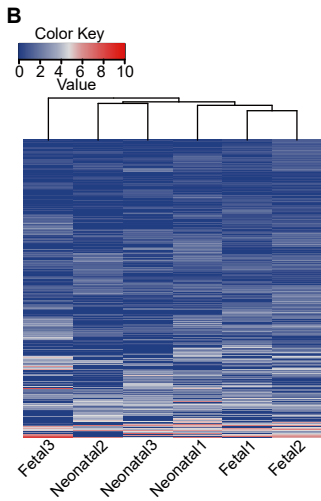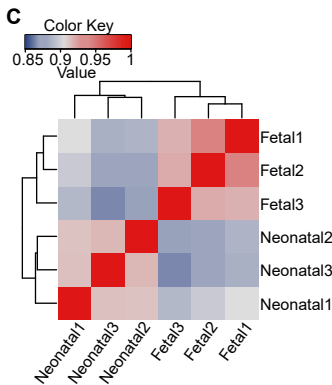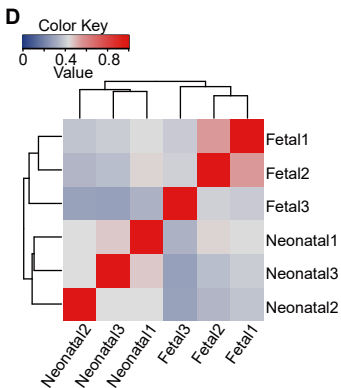

Supplement: Supplementary file 1 [file genes-09-00443-s001.zip › Fig.S2.pdf]

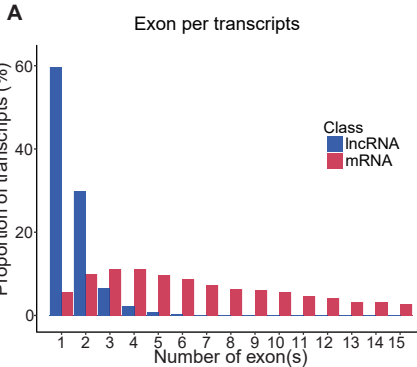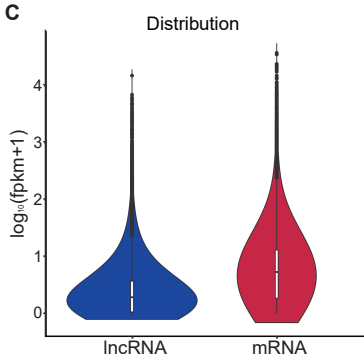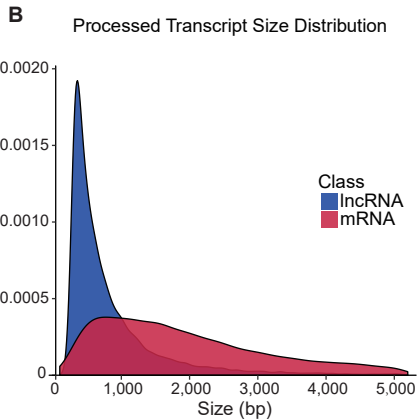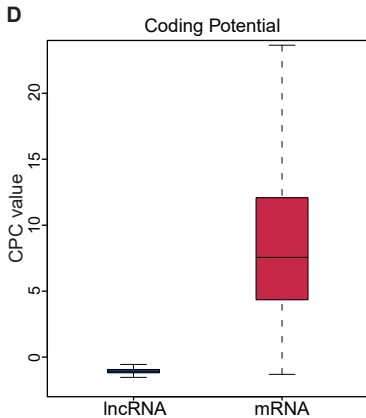

Supplement: Supplementary file 1 [file genes-09-00443-s001.zip › Fig.S3.pdf]
